# Modelling the coefficient of thermal expansion in graphite crystals: implications of lattice strain due to irradiation and pressure

**DOI:** 10.1098/rspa.2018.0075

**Published:** 2018-10-31

**Authors:** Barry Marsden, Andrew Mummery, Paul Mummery

**Affiliations:** 1 Nuclear Graphite Research Group, School of Mechanical, Aerospace and Civil Engineering, University of Manchester, Manchester M13 9PL, UK; 2 Wadham College, University of Oxford, Parks Road, Oxford OX1 3PN, UK

**Keywords:** modelling, thermal expansion, graphite crystal, irradiation, pressure, polycrystalline graphite

## Abstract

Theoretical models for the coefficient of thermal expansion (CTE) first proposed in the 1970s are expanded upon, allowing them, for the first time, to be implemented over a wide temperature range. The models are of interest because they predict the effects of the changes in the crystal lattice spacing and crystallite modulus on the CTE. Hence, they can in turn be used to investigate the influence of pressure and irradiation on the CTE. To date, typographical and mathematical errors and incomplete or conflicting assumptions between the various papers had made the complex mathematical formulations difficult, if not impossible, to follow and apply. This paper has two main aims: firstly to revisit and review the CTE models, correcting the errors and compiling and updating various input data, secondly to use the revised models to investigate the effect of loading and irradiation on the CTE. In particular, the models have been applied to data for natural and highly orientated pyrolytic graphite and compared with experimental data, giving an insight into the influence of temperature, loading and irradiation on both single crystal and polycrystalline graphite. The findings lend credence to postulated microstructural mechanisms attributed to the in-reactor behaviour of nuclear graphite, which finds a wide use in predictive multiscale modelling.

## 1. Introduction

Significant property and dimensional changes are exhibited by polycrystalline graphite subjected to fast neutron irradiation [1] and high pressure [2]. Of particular interest are the changes to the coefficient of thermal expansion (CTE). It is postulated that changes to the CTE in polycrystalline graphite under loading and irradiation are related to a combination of changes to: the crystallites, the porosity, the crystal orientation, the microstructural stiffness or more likely a combination of these factors [3,4]. To separate out these effects, it is therefore important to understand and define the possible contribution that crystal CTE changes have on the bulk CTE. To this end, Kelly [5] derived several theoretical models capable of predicting the CTE of a graphite crystal as a function of temperature and crystal lattice spacing [6–13]. Unfortunately, to date, these theoretical models have not been widely taken up as typographical errors and incomplete or conflicting assumptions between the various papers make the complex mathematical formulations difficult, if not impossible, to follow and apply.

This paper revisits and reviews these CTE models, correcting the errors as well as compiling and updating various model input data. The models are used to investigate the effect of loading and irradiation on the CTE, and are compared with available experimental data. In particular, the models have been applied to data for natural and highly orientated pyrolytic graphite and compared with experimental data, giving an insight into the influence of temperature, loading and irradiation on both single crystal and polycrystalline graphite.

## 2. Development of a graphite semi-continuum coefficient of thermal expansion model

**Figure 1.**
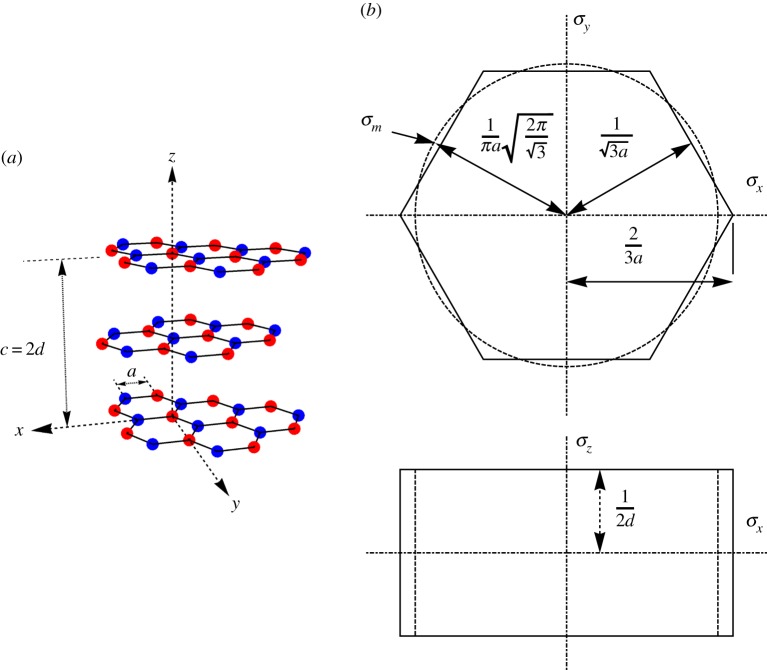
(*a*) Neighbouring basal layers of graphite; coloured atoms emphasize the offset between ABAB basal planes. (*b*) Graphite crystal lattice and first Brillouin zone, *a* = 0.246 nm, *d* = 0.335 nm [14,15]; the ‘equivalent cylinder’ is shown dashed. (Online version in colour.)

The relationship between crystal strains *e*_*ij*_ and crystal stresses *τ*_*ij*_ are defined by the Cartesian co-ordinate system, given in figure 1*a*, with the *z*-axis parallel to the hexagonal axis of the crystal *c*-axis as follows:

2.1 $$\left(\begin{array}{c} e_{xx}\\ e_{yy}\\ e_{zz}\\ e_{zx}\\ e_{zy}\\ e_{xy} \end{array}\right)=\left(\begin{array}{cccccc} S_{11} & S_{12} & S_{13} & 0 & 0 & 0\\ S_{12} & S_{11} & S_{13} & 0 & 0 & 0\\ S_{13} & S_{13} & S_{33} & 0 & 0 & 0\\ 0 & 0 & 0 & S_{44} & 0 & 0\\ 0 & 0 & 0 & 0 & S_{44} & 0\\ 0 & 0 & 0 & 0 & 0 & \frac{S_{11}-S_{12}}{2} \end{array}\right)\left(\begin{array}{c} \tau_{xx}\\ \tau_{yy}\\ \tau_{zz}\\ \tau_{zx}\\ \tau_{zy}\\ \tau_{xy} \end{array}\right).$$

The stresses *τ*_*ij*_ are defined as the force acting on unit area parallel to the *i*th direction, the normal to the unit area being in the *j*th direction. The matrix elements, *S*_*ij*_, are the elastic compliance constants. This matrix can be inverted to obtain the elastic stiffness constants, *C*_*ij*_, as given below:

2.2 $$\left(\begin{array}{c} \tau_{xx}\\ \tau_{yy}\\ \tau_{zz}\\ \tau_{zx}\\ \tau_{zy}\\ \tau_{xy} \end{array}\right)=\left(\begin{array}{cccccc} C_{11} & C_{12} & C_{13} & 0 & 0 & 0\\ C_{12} & C_{11} & C_{13} & 0 & 0 & 0\\ C_{13} & C_{13} & C_{33} & 0 & 0 & 0\\ 0 & 0 & 0 & C_{44} & 0 & 0\\ 0 & 0 & 0 & 0 & C_{44} & 0\\ 0 & 0 & 0 & 0 & 0 & \frac{C_{11}-C_{12}}{2} \end{array}\right)\left(\begin{array}{c} e_{xx}\\ e_{yy}\\ e_{zz}\\ e_{zx}\\ e_{zy}\\ e_{xy} \end{array}\right).$$

The relationships between the compliance and stiffness constants are:

$$\begin{array}{rl} S_{11} & =\frac{C_{11}C_{33}-C_{13}^{2}}{(C_{11}-C_{12})X},\\ S_{33} & =\frac{C_{11}+C_{12}}{X},\\ S_{13} & =-\frac{C_{13}}{X},\\ S_{12} & =\frac{C_{13}^{2}-C_{12}C_{33}}{(C_{11}-C_{12})X},\\ S_{44} & =\frac{1}{C_{44}},\\ S_{66} & =\frac{2}{C_{11}-C_{12}}, \end{array}$$

where

2.3 $$X=C_{33}(C_{11}+C_{12})-2C_{13}^{2}.$$

The stiffness constants for a graphite crystal are given in table 1 of appendix A; these were obtained using Born's long-wave method [14] and local density approximation (LDA) [15]. Owing to the crystal structure in which atoms within layers are held together by strong covalent bonding, whereas bonding between layers is via weak van der Waals bonds, the values of the stiffness constants perpendicular, *C*_33_, and parallel, *C*_11_, to the basal planes are different by orders of magnitude. The shear components *C*_44_ and *C*_66_ = (*C*_11_ − *C*_12_)/2 are also small.

**Table 1.** Crystal elastic stiffness and compliance constants for graphite.

stiffness constants [14,15]	GPa	compliance constants (calculated from stiffness constants)	10^−12^ (Pa)^−1^
*C*_11_	1060	*S*_11_	0.97
*C*_33_	36.5	*S*_33_	27.47
*C*_12_	180	*S*_12_	-0.17
*C*_13_	7.9	*S*_13_	-0.18
*C*_44_	5.05	*S*_44_	198
*C*_66_	440	*S*_66_	2.27

The most thermodynamically stable form of graphite crystal is the ABAB hexagonal stacking, giving a theoretical density of 2.266 × 10^3^ kg m^−3^. The lattice *a*-spacing is 0.1415 nm and the *c*-spacing is 0.335 nm—calculated using LDA [15]; the lattice *d*-spacing is half that of the *c*-spacing.

The development of a semi-continuum model is based on the consideration of the free energy, *F*, in a unit cube of graphite crystallites in a thermally dilated state, assuming that there are no shear strains as follows:

2.4 $$\begin{array}{rl} F & =U_{0}+\frac{1}{2}(e_{xx}^{2}+e_{yy}^{2})C_{11}+\frac{1}{2}C_{33}e_{zz}^{2}+C_{12}e_{xx}e_{yy}+C_{13}e_{zz}(e_{xx}+e_{yy})\\ & \;+kT{∭}_{BZ}\sum_{p}\ln\left[1-\exp\left(-\frac{hv_{p}}{kT}\right)\right]d^{3}\sigma , \end{array}$$

where the first term, *U*_0_, is the energy/unit volume at absolute zero temperature in the unrestrained state; the next four terms are the elastic strain energy density; and the last term is the thermal energy density of the first Brillouin zone, where

*v*_*p*_ is the frequency of the *p*th vibrational mode (Hz),
*k* is Boltzmann's constant (1.38064852 × 10^−23^ J K^−1^),
*h* is Planck's constant (6.626070040 × 10^−34^ J s^−1^), and
*T* is the absolute temperature (K).

The integral is over the first Brillouin zone of the graphite crystal structure [16,17], i.e. the reciprocal lattice space between two adjacent basal planes; see figure 1*b*. Note that there must be a whole number of waves, denoted by the wavenumber, in the *x* and *y* planes. Therefore, the wavenumber *z* varies from ±1/2*d* =  ± 1.49 × 10^9^ m^−1^. To simplify the integration, the hexagonal prismatic Brillouin zone has been approximated as a cylinder with an equivalent radius, defined as:

2.5 $$\sigma_{m}=\frac{1}{\pi a}\sqrt{\frac{2\pi }{\sqrt{3}}}.$$

We use the short-hand notation *d*^3^*σ*≡*dσ*_*x*_*dσ*_*y*_*dσ*_*z*_.

The integral will be performed in cylindrical co-ordinates (*σ*_*a*_, *ϕ*, *σ*_*z*_) with *σ*_*a*_ defined as $\sigma_{a}=\sqrt{\sigma_{x}^{2}+\sigma_{y}^{2}}$, tranforming to cylindrical co-ordinates and assuming cylindrical symmetry leads to *d*^3^*σ* = 2*πσ*_*a*_*dσ*_*a*_*dσ*_*z*_.

It should be noted that previous authors [9] did not include the integral over the Brillouin zone at this stage of the derivation. In this paper, we explicitly separate the summation over frequencies from the summation over modes (integration over the first Brillouin zone) for clarity. This also removes confusion about the dimensionality of the different terms in equation (2.4).

The principal graphite crystallite coefficients of thermal expansion required are given by the differentials

2.6 $$\begin{array}{rl} \alpha_{c} & =\frac{\partial e_{zz}}{\partial T}\\ \;\mathrm{and}\;\alpha_{a} & =\frac{\partial e_{xx}}{\partial T}=\frac{\partial e_{yy}}{\partial T}. \end{array}\}$$

At equilibrium, the free energy, *F*, given by equation (2.4) is a minimum with respect to the strains. Thus, noting that *v*_*p*_ is a function of strain, differentiating equation (2.4) with respect to *e*_*zz*_ and *e*_*xx*_ and equating each to zero leads to

2.7 $$\begin{array}{rl} & e_{xx}^{2}\frac{\partial C_{11}}{\partial e_{zz}}+C_{33}e_{zz}+\frac{1}{2}e_{zz}^{2}\frac{\partial C_{33}}{\partial e_{zz}}+e_{xx}^{2}\frac{\partial C_{12}}{\partial e_{zz}}+2e_{xx}C_{13}+2e_{zz}e_{xx}\frac{\partial C_{33}}{\partial e_{zz}}\\ & \;-{∭}_{BZ}\sum_{p}\frac{hv_{p}}{\exp(hv_{p}/kT)-1}{\left[\gamma_{p}\right]}_{zz}\;d^{3}\sigma =0\\ \;\mathrm{and}\; & e_{xx}^{2}\frac{\partial C_{11}}{\partial e_{xx}}+(C_{11}+C_{12})e_{xx}+\frac{1}{2}e_{zz}^{2}\frac{\partial C_{33}}{\partial e_{xx}}+e_{xx}^{2}\frac{\partial C_{12}}{\partial e_{xx}}+e_{zz}C_{13}+2e_{zz}e_{xx}\frac{\partial C_{33}}{\partial e_{xx}}\\ & \;-{∭}_{BZ}\sum_{p}\frac{hv_{p}}{\exp(hv_{p}/kT)-1}{\left[\gamma_{p}\right]}_{xx}\;d^{3}\sigma =0, \end{array}\}$$

where [*γ*_*p*_]_*ii*_≡ − (1/*v*_*p*_)(∂*v*_*p*_/∂*e*_*ii*_), which are the mode Grüneisen [18] parameters describing the frequency shifts due to strain which have previously been referred to as the anharmonic terms. In addition, the equations have been simplified by using the fact that, due to symmetry, the strains in the crystal basal planes are equal, i.e. *e*_*xx*_ = *e*_*yy*_.

It will be shown analytically later that the derivatives of the elastic constants are of greater magnitude than the elastic constants themselves; however, the elastic strains are very small, and so terms that are higher order in the elastic strains can be neglected. Thus, equation (2.7) can be simplified to

2.8 $$\begin{array}{rl} & C_{33}e_{zz}+2e_{xx}C_{13}-{∭}_{BZ}\sum_{p}\frac{hv_{p}}{\exp(hv_{p}/kT)-1}{\left[\gamma_{p}\right]}_{zz}\;d^{3}\sigma =0\\ \;\mathrm{and}\; & (C_{11}+C_{12})e_{xx}+e_{zz}C_{13}-{∭}_{BZ}\sum_{p}\frac{hv_{p}}{\exp(hv_{p}/kT)-1}{\left[\gamma_{p}\right]}_{xx}d^{3}\sigma =0. \end{array}\}$$

Previously, the assumption was made that the higher order strain terms given above are negligible, although this is not discussed in [9].

Equations (2.8) can be solved for *e*_*xx*_ and *e*_*yy*_ and then differentiated with respect to temperature to give the principal crystal CTE as

2.9 $$\begin{array}{rl} \frac{\partial e_{zz}}{\partial T} & =\alpha_{c}=\left(\frac{C_{11}+C_{12}}{C_{33}(C_{11}+C_{12})-2C_{13}^{2}}\right){∭}_{BZ}\sum_{p}k{\left(\frac{hv_{p}}{kT}\right)}^{2}\frac{\exp(hv_{p}/kT){\left[\gamma_{p}\right]}_{zz}}{{\left\{\exp(hv_{p}/kT)-1\right\}}^{2}}d^{3}\sigma \\ & \;-\left(\frac{2C_{13}}{C_{33}(C_{11}+C_{12})-2C_{13}^{2}}\right){∭}_{BZ}\sum_{p}k{\left(\frac{hv_{p}}{kT}\right)}^{2}\frac{\exp(hv_{p}/kT){\left[\gamma_{p}\right]}_{xx}}{{\left\{\exp(hv_{p}/kT)-1\right\}}^{2}}d^{3}\sigma \end{array}$$

and

2.10 $$\begin{array}{rl} \frac{\partial e_{xx}}{\partial T} & =\alpha_{a}=\left(\frac{C_{33}}{C_{33}(C_{11}+C_{12})-2C_{13}^{2}}\right){∭}_{BZ}\sum_{p}k{\left(\frac{hv_{p}}{kT}\right)}^{2}\frac{\exp(hv_{p}/kT){\left[\gamma_{p}\right]}_{xx}}{{\left\{\exp(hv_{p}/kT)-1\right\}}^{2}}d^{3}\sigma \\ & \;-\left(\frac{C_{13}}{C_{33}(C_{11}+C_{12})-2C_{13}^{2}}\right){∭}_{BZ}\sum_{p}k{\left(\frac{hv_{p}}{kT}\right)}^{2}\frac{\exp(hv_{p}/kT){\left[\gamma_{p}\right]}_{zz}}{{\left\{\exp(hv_{p}/kT)-1\right\}}^{2}}d^{3}\sigma . \end{array}$$

The elastic stiffness constants, *C*_*ij*_, can be related to the elastic compliance constants, *S*_*ij*_, by equations (2.3), thus equations (2.9) and (2.10) become

2.11 $$\begin{array}{rl} \alpha_{c} & =S_{33}{∭}_{BZ}\sum_{p}k{\left(\frac{hv_{p}}{kT}\right)}^{2}\frac{\exp(hv_{p}/kT){\left[\gamma_{p}\right]}_{zz}}{{\left\{\exp(hv_{p}/kT)-1\right\}}^{2}}d^{3}\sigma \\ & \;+2S_{13}{∭}_{BZ}\sum_{p}k{\left(\frac{hv_{p}}{kT}\right)}^{2}\frac{\exp(hv_{p}/kT){\left[\gamma_{p}\right]}_{xx}}{{\left\{\exp(hv_{p}/kT)-1\right\}}^{2}}d^{3}\sigma \end{array}$$

and

2.12 $$\begin{array}{rl} \alpha_{a} & =(S_{11}+S_{12}){∭}_{BZ}\sum_{p}k{\left(\frac{hv_{p}}{kT}\right)}^{2}\frac{\exp(hv_{p}/kT){\left[\gamma_{p}\right]}_{xx}}{{\left\{\exp(hv_{p}/kT)-1\right\}}^{2}}d^{3}\sigma \\ & \;+S_{13}{∭}_{BZ}\sum_{p}k{\left(\frac{hv_{p}}{kT}\right)}^{2}\frac{\exp(hv_{p}/kT){\left[\gamma_{p}\right]}_{zz}}{{\left\{\exp(hv_{p}/kT)-1\right\}}^{2}}d^{3}\sigma . \end{array}$$

A model for the frequencies of crystallite vibration modes, *v*_*p*_, is provided by Komatsu & Nagamiya [19,20], as described in the next section.

## 3. Graphite vibration model

To solve for *α*_*c*_ and *α*_*a*_, we require the frequency of the vibrational modes in the longitudinal, transverse and out-of-plane principal directions and also the anharmonic modes — themselves functions of the principal vibrational frequencies. Below, we summarize the Komatsu [20] model for calculating the principal frequencies; this assumes that the graphite crystallites can be modelled as elastic extensional vibrations in a series of *n* thin plates [21,22]. The present authors modify Komatsu's notation [20] to match the rest of this paper. The equations for the elastic extensional vibrations in a series of *n* thin plates are

3.1 $$\begin{array}{rl} \frac{\partial^{2}u_{n}}{\partial t^{2}} & =\frac{1}{\rho (1-\nu^{2})}\left\{C_{11}\frac{\partial^{2}u_{n}}{\partial x^{2}}+\frac{C_{11}-C_{12}}{2}(1-\nu )\frac{\partial^{2}u_{n}}{\partial y^{2}}+\frac{C_{11}-C_{12}}{2}(1-\nu )\frac{\partial^{2}v_{n}}{\partial x\partial y}\right\},\\ \frac{\partial^{2}v_{n}}{\partial t^{2}} & =\frac{1}{\rho (1-\nu^{2})}\left\{C_{11}\frac{\partial^{2}v_{n}}{\partial y^{2}}+\frac{C_{11}-C_{12}}{2}(1-\nu )\frac{\partial^{2}v_{n}}{\partial x^{2}}+\frac{C_{11}-C_{12}}{2}(1-\nu )\frac{\partial^{2}u_{n}}{\partial x\partial y}\right\}\\ \;\mathrm{and}\;\frac{\partial^{2}w_{n}}{\partial t^{2}} & =-\delta^{2}\left[\frac{\partial^{4}w_{n}}{\partial x^{4}}+2\frac{\partial^{4}w_{n}}{\partial x^{2}\partial y^{2}}+\frac{\partial^{4}w_{n}}{\partial y^{4}}\right]+\frac{C_{33}}{\rho d^{2}}(w_{n+1}+w_{n-1}-2w_{n}), \end{array}\}$$

where *u*_*n*_, *v*_*n*_, *w*_*n*_ are the displacements at a lattice layer point (*x*, *y*) in the *x*-, *y*- and *z*-axis directions, differentiated with respect to time, *t*; *ν* is Poisson's ratio, *ρ* is the density and *d* is the graphite interlayer spacing; *δ* is related to the bending modulus of a plate. Kelly & Walker [9] give a value of *δ* = 6.11 × 10^−7^ m^2^ s^−1^. In the third equation of (3.1), the second term on the r.h.s. accounts for the interaction between neighbouring plates.

Komatsu [20] modified equations (3.1) to account for shear stress, obtaining the following terms:

3.2 $$\begin{array}{rl} \frac{\partial^{2}u_{n}}{\partial t^{2}} & =\frac{1}{\rho (1-\nu^{2})}\left\{C_{11}\frac{\partial^{2}u_{n}}{\partial x^{2}}+\frac{C_{11}-C_{12}}{2}(1-\nu )\frac{\partial^{2}u_{n}}{\partial y^{2}}+\frac{C_{11}-C_{12}}{2}(1-\nu )\frac{\partial^{2}v_{n}}{\partial x\partial y}\right\}\\ & \;+\frac{C_{44}}{\rho d^{2}}(u_{n+1}+u_{n-1}-2u_{n})+\frac{C_{44}}{2\rho d}\left(\frac{\partial w_{n+1}}{\partial x}-\frac{\partial w_{n-1}}{\partial x}\right),\\ \frac{\partial^{2}v_{n}}{\partial t^{2}} & =\frac{1}{\rho (1-\nu^{2})}\left\{C_{11}\frac{\partial^{2}v_{n}}{\partial y^{2}}+\frac{C_{11}-C_{12}}{2}(1-\nu )\frac{\partial^{2}v_{n}}{\partial x^{2}}+\frac{C_{11}-C_{12}}{2}(1-\nu )\frac{\partial^{2}u_{n}}{\partial x\partial y}\right\}\\ & \;+\frac{C_{44}}{\rho d^{2}}(v_{n+1}+v_{n-1}-2v_{n})+\frac{C_{44}}{2\rho d}\left(\frac{\partial w_{n+1}}{\partial y}-\frac{\partial w_{n-1}}{\partial y}\right)\\ \;\mathrm{and}\;\frac{\partial^{2}w_{n}}{\partial t^{2}} & =-\delta^{2}\left[\frac{\partial^{4}w_{n}}{\partial x^{4}}+2\frac{\partial^{4}w_{n}}{\partial x^{2}\partial y^{2}}+\frac{\partial^{4}w_{n}}{\partial y^{4}}\right]+\frac{C_{33}}{\rho d^{2}}(w_{n+1}+w_{n-1}-2w_{n})\\ & \;+\frac{C_{44}}{\rho }\left(\frac{\partial^{2}w_{n}}{\partial x^{2}}+\frac{\partial^{2}w_{n}}{\partial y^{2}}\right)+\frac{C_{44}}{2\rho d}\left(\frac{\partial u_{n+1}}{\partial x}-\frac{\partial u_{n-1}}{\partial x}+\frac{\partial v_{n+1}}{\partial y}-\frac{\partial v_{n-1}}{\partial y}\right). \end{array}\}$$

The longitudinal (transverse) velocity is defined as the square root of the pre-factor in front of the *x* (*y*) second derivative term in the *u*_*n*_ displacement equation. Assuming that Poisson's ratio is zero, the velocity of the longitudinal wave, *V*_L_, can be simplified to

3.3 $$V_{L}=\sqrt{\frac{C_{11}}{\rho }}.$$

Similarly, the velocity of the transverse wave *V*_T_ is given by

3.4 $$V_{T}=\sqrt{\frac{C_{11}-C_{12}}{2\rho }}.$$

Equations (3.2) are solved using the conventional substitution in reciprocal space, i.e. in the first Brilliouin zone

3.5 $$\begin{array}{rl} u_{n} & =U\exp\{2\pi i(\sigma_{x}x+\sigma_{y}y+\sigma_{z}nd-v_{U}t)\},\\ v_{n} & =V\exp\{2\pi i(\sigma_{x}x+\sigma_{y}y+\sigma_{z}nd-v_{V}t)\}\\ \;\mathrm{and}\;w_{n} & =W\exp\{2\pi i(\sigma_{x}x+\sigma_{y}y+\sigma_{z}nd-v_{W}t)\}, \end{array}\}$$

where *σ*_*x*_, *σ*_*y*_ and *σ*_*z*_ are the wavenumbers as discussed previously and *v*_*U*_, *v*_*V*_ and *v*_*W*_ are frequencies of the vibrational mode. Komatsu [20] solved for the principal vibrational modes, *v*_*p*_, which are linear superpositions of *v*_*U*_, *v*_*V*_ and *v*_*W*_, leading to

3.6 $$\begin{array}{rl} v_{1} & =\sqrt{\frac{C_{11}\sigma_{a}^{2}}{\rho }+\frac{C_{44}}{\rho \pi^{2}d^{2}}\sin^{2}(\pi \sigma_{z}d)},\\ v_{2} & =\sqrt{\frac{(C_{11}-C_{12})\sigma_{a}^{2}}{2\rho }+\frac{C_{44}}{\rho \pi^{2}d^{2}}\sin^{2}(\pi \sigma_{z}d)}\\ \;\mathrm{and}\;v_{3} & =\sqrt{4\pi^{2}\delta^{2}\sigma_{a}^{4}+\frac{C_{33}}{\rho \pi^{2}d^{2}}\sin^{2}(\pi d\sigma_{z})+\frac{C_{44}\sigma_{a}^{2}}{\rho }}, \end{array}\}$$

where again *σ*^2^_*a*_ = *σ*^2^_*x*_ + *σ*^2^_*y*_. It should be noted that, in the derivation, Komatsu [20] assumes that *τ* = *C*_44_/*ρ* is small and therefore cross terms with *τ* as a pre-factor are ignored.

**Figure 2.**
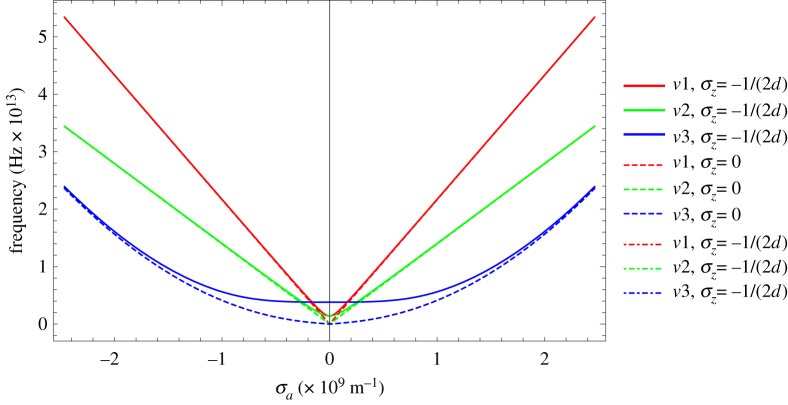
Examples of vibration modes plotted over the first Brillouin zone (frequency *ν*_*i*_ versus Brillouin radius *σ*_*a*_). Note for each vibration mode the curves overlap except for *v*_3_, where the difference is small. (Online version in colour.)

The three vibration modes given in equations (3.6) are insensitive to *σ*_*z*_. For illustration, three of the vibration modes are plotted in figure 2 over the first Brillouin zone for different values of *σ*_*z*_.

Equations (3.6) are the equations for lattice vibration used previously [9]. The anharmonic functions for the longitudinal, transverse and out-of-plane principal modes are as follows:

3.7 $$\begin{array}{rl} {[\gamma_{1}]}_{ii} & =-\frac{1}{v_{1}}\frac{\partial v_{1}}{\partial e_{ii}}=-\frac{1}{2}{\left[\frac{C_{11}\sigma_{a}^{2}}{\rho }+\frac{C_{44}}{\rho \pi^{2}d^{2}}\sin^{2}(\pi \sigma_{z}d)\right]}^{-1}\\ & \;\times \left\{\frac{\sigma_{a}^{2}}{\rho }\frac{\partial C_{11}}{\partial e_{ii}}+\frac{\sin^{2}(\pi \sigma_{z}d)}{\rho \pi^{2}d^{2}}\frac{\partial C_{44}}{\partial e_{ii}}\right\},\\ {\left[\gamma_{2}\right]}_{ii} & =-\frac{1}{v_{2}}\frac{\partial v_{2}}{\partial e_{ii}}=-\frac{1}{2}{\left[\frac{(C_{11}-C_{12})\sigma_{a}^{2}}{2\rho }+\frac{C_{44}}{\rho \pi^{2}d^{2}}\sin^{2}(\pi \sigma_{z}d)\right]}^{-1}\\ & \;\times \left\{\frac{\sigma_{a}^{2}}{2\rho }\frac{\partial (C_{11}-C_{12})}{\partial e_{ii}}+\frac{\sin^{2}(\pi \sigma_{z}d)}{\rho \pi^{2}d^{2}}\frac{\partial C_{44}}{\partial e_{ii}}\right\}\\ \;\mathrm{and}\;{\left[\gamma_{3}\right]}_{ii} & =-\frac{1}{v_{3}}\frac{\partial v_{3}}{\partial e_{ii}}=-\frac{1}{2}{\left[4\pi^{2}\delta^{2}\sigma_{a}^{4}+\frac{C_{33}}{\rho \pi^{2}d^{2}}\sin^{2}(\pi d\sigma_{z})+\frac{C_{44}\sigma_{a}^{2}}{\rho }\right]}^{-1}\\ & \;\times \left\{8\pi^{2}\delta \sigma_{a}^{4}\frac{\partial \delta }{\partial e_{ii}}+\frac{\sin^{2}(\pi d\sigma_{z})}{\rho \pi^{2}d^{2}}\frac{\partial C_{33}}{\partial e_{ii}}+\frac{\sigma_{a}^{2}}{\rho }\frac{\partial C_{44}}{\partial e_{ii}}\right\}. \end{array}\}$$

To evaluate these, the differentials of the elastic constants ∂*C*_*ij*_/∂*e*_*kk*_ and ∂*δ*/∂*e*_*ii*_ are required with respect to the strain in the longitudinal and transverse in-plane *x*-axis and the out-of-plane *z*-axis. The values used in our simulations are given in table 2 (appendix A). In this paper, only the anharmonic functions [*γ*_3_]_*zz*_, in their full form, are used in the derived expressions for *α*_*c*_. The in-plane [*γ*_1_]_*xx*_ and [*γ*_2_]_*xx*_ anharmonics are simplified in the case of *α*_*a*_, and in effect can be regarded as constants.

**Table 2.** Estimated values of derivatives required to evaluate the anharmonic functions.

differential	value	reference
$\frac{\partial C_{33}}{\partial e_{zz}}$	−6 × 10^11^ N m^−2^	[7]
	−5.5 × 10^11^ N m^−2^	[55]
$\frac{\partial C_{44}}{\partial e_{zz}}$	−3.55 × 10^10^ N m^−2^	[9]
	−10 × 10^10^ N m^−2^	[9]
	−8.1 × 10^10^ N m^−2^	[8]
	−4.05 × 10^10^ N m^−2^	[7]
	−2.4 × 10^10^ N m^−2^	[55]
$\frac{\partial \delta }{\partial e_{zz}}$	−1.4 × 10^−7^ m^2^ s^−1^	[9,56]
$\frac{1}{C_{11}}\frac{\partial C_{11}}{\partial e_{xx}}$	−8.2	[6]
$\frac{1}{\left(C_{11}\;-\;C_{12}\right)}\frac{\partial (C_{11}\;-\;C_{12})}{\partial e_{xx}}$	−8.2	[6]
$\frac{1}{\delta }\frac{\partial \delta }{\partial e_{xx}}$	8.2	[6]

**Figure 3.**
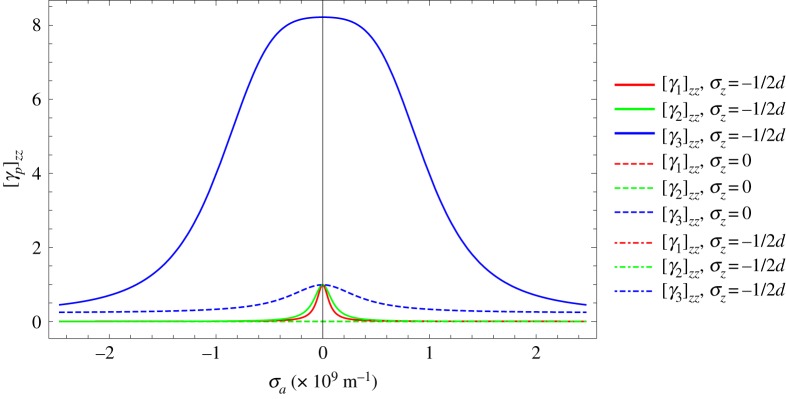
Anharmonic functions [*γ*_*p*_]_*zz*_ versus Brillouin radius *σ*_*a*_ for different values of *σ*_*z*_. Note the largest contribution to CTE is from [*γ*_3_]_*zz*_ for *σ*_*z*_ = ± 1/2*d*, the centre-top and centre-bottom of the Brillouin zone, with the smallest contribution when *σ*_*z*_ = 0. (Online version in colour.)

The anharmonic functions [*γ*_*p*_]_*zz*_ are plotted as a function of *σ*_*a*_ for different values of *σ*_*z*_ in figure 3.

The differentials of the anharmonic functions with respect to *e*_*xx*_ are discussed later with reference to the derivation of *α*_*a*_.

## 4. Coefficient of thermal expansion perpendicular to the basal plane—*α*_*c*_

Having obtained expressions for the orthogonal vibrations and anharmonic modes, expressions for the CTE can be derived using the semi-continuum model over a range of temperatures to compare the model with experimental data. Firstly, we consider the thermal expansion coefficient perpendicular to the basal plane.

It can be seen from table 1 (appendix A) that *S*_13_ is two orders of magnitude smaller than *S*_33_, thus equation (2.11) simplifies to

4.1 $$\alpha_{c}=S_{33}{∭}_{BZ}\sum_{p}k{\left(\frac{hv_{p}}{kT}\right)}^{2}\frac{\exp(hv_{p}/kT){\left[\gamma_{p}\right]}_{zz}}{{\left\{\exp(hv_{p}/kT)-1\right\}}^{2}}d^{3}\sigma .$$

There are three possible vibration modes to consider when calculating *α*_*c*_: in-plane longitudinal mode, in-plane transverse mode and out-of-plane mode. Previously it has been argued that only the out-of-plane mode is significant. Unfortunately, the reasoning behind this assumption in [9] is unclear and there appear to be mistakes in the references and supporting data. However, due to the large difference in stiffness between the in-plane *C*_11_ (1060 GPa) and the out-of-plane *C*_33_ (36.5 GPa) it is not unreasonable to assume that the out-of-plane strain *e*_*zz*_ would have less influence on the values of *C*_11_ and *C*_12_ than it would have on *C*_33_. This assumption appears to be justified by the implementation of the theory as demonstrated below. The final step is to perform the integral over a cylinder of equivalent volume to the true Brillouin zone,

4.2 $$\begin{array}{rl} \alpha_{c} & =-\pi S_{33}k{\left(\frac{h}{kT}\right)}^{2}\int_{0}^{\sigma_{m}}\int_{-1/2d}^{1/2d}\\ & \;\times \frac{\sigma_{a}\{8\pi^{2}\delta \sigma_{a}^{4}(\partial \delta /\partial e_{zz})+\left(\sin^{2}(\pi d\sigma_{z})/\rho \pi^{2}d^{2})(\partial C_{33}/\partial e_{zz}\right)+\left(\sigma_{a}^{2}/\rho )(\partial C_{44}/\partial e_{zz}\right)\}}{{\left\{\exp(h/kT\sqrt{4\pi^{2}\delta^{2}\sigma_{a}^{4}+(C_{33}/\rho \pi^{2}d^{2})\sin^{2}(\pi d\sigma_{z})+C_{44}\sigma_{a}^{2}/\rho })-1\right\}}^{2}}\\ & \;\times \exp\left(\frac{h}{kT}\sqrt{4\pi^{2}\delta^{2}\sigma_{a}^{4}+\frac{C_{33}}{\rho \pi^{2}d^{2}}\sin^{2}(\pi d\sigma_{z})+\frac{C_{44}\sigma_{a}^{2}}{\rho }}\right)d\sigma_{z}\;d\sigma_{a}. \end{array}$$

Data giving the changes in graphite crystal CTE as a function of temperature can be obtained by direct thermal expansion measurements or via changes to lattice spacing using X-ray diffraction (XRD). Measurements have been obtained using either naturally occurring graphite crystals or highly oriented pyrolytic graphite (HOPG). The quality of these measurements depends on the graphitic perfection of the sample used, which is usually defined using a p-factor [23,24]; p-factors range from zero to unity, representing the degree of cryptographic order. Measurements made on various graphitic structures with p-factors between 0 and 0.7, including irradiated graphite by Steward *et al.* [25,26], show that curves of changed interlayer *d*-spacing versus temperature are parallel to one another between ∼0 and ∼2600 K, indicating that the thermal expansion coefficients, which are related to the slopes of these curves, are unaffected by significant disorder of the graphite lattice. This observation agrees with the estimates of change in CTE due to strain and irradiation discussed later in this paper.

Morgan [27] presents a comprehensive set of XRD measurements giving changes to *α*_*a*_ and *α*_*c*_ taken from Kellett & Richards [28], Nelson & Riley [29], Yang [30], Kellett *et al.* [31] and Baskin & Meyer [32].

**Figure 4.**
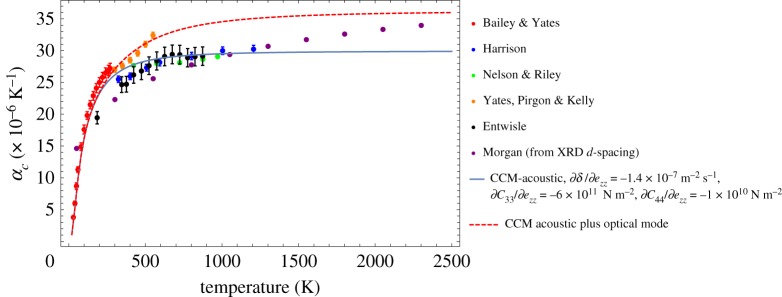
Comparison of fit of the theoretical, semi-continuum model with experimental data; see [27,29,34–37]. (Online version in colour.)

Kelly [33] also presents a dataset of crystal CTE measurements, but there is confusion with regard to the source of some of the data. Figure 4 brings together various datasets for *α*_*c*_ from Entwisle [34], Bailey & Yates [35], Yates *et al*. [36], Harrison [37], Nelson & Riley [29] and Morgan [27]. Where the measurement error on data are available error bars are given; most of the data originates from XRD data on graphitic materials with a p-factor of 0.2. The prediction of *α*_*c*_ using equation (4.2) is also included. While equation (4.2) fits well at low values to medium values of temperature, it does not appear to capture the measured CTE increase at higher temperatures. Also plotted is an attempt to include the contribution to the CTE of the optical modes of the system. It is possible to make the simplifying assumption [6] that extending the upper integration limit to $\sqrt{2}\sigma_{m}$ has the effect of including contributions from both the in-plane acoustic and the higher frequency optical mode. It is difficult to say with current experimental data whether the inclusion of optical modes produces a better fit.

The behaviour of the thermal expansion coefficient can be understood in the high and low temperature limits by examining the leading order behaviour for *hv*_3_≫*kT* and *hv*_3_≪*kT*.

For low temperatures, *hv*_3_≫*kT*, the leading order behaviour is

4.3 $$\begin{array}{rl} \alpha_{c} & \approx -\pi S_{33}k{\left(\frac{h}{kT}\right)}^{2}\int_{0}^{\sigma_{m}}\int_{-1/2d}^{1/2d}\sigma_{a}\left\{8\pi^{2}\delta \sigma_{a}^{4}\frac{\partial \delta }{\partial e_{zz}}+\frac{\sin^{2}(\pi d\sigma_{z})}{\rho \pi^{2}d^{2}}\frac{\partial C_{33}}{\partial e_{zz}}+\frac{\sigma_{a}^{2}}{\rho }\frac{\partial C_{44}}{\partial e_{zz}}\right\}\\ & \;\times \exp\left(-\frac{h}{kT}\sqrt{4\pi^{2}\delta^{2}\sigma_{a}^{4}+\frac{C_{33}}{\rho \pi^{2}d^{2}}\sin^{2}(\pi d\sigma_{z})+\frac{C_{44}\sigma_{a}^{2}}{\rho }}\right)d\sigma_{z}\;d\sigma_{a}. \end{array}$$

For high temperatures, *hv*_3_≪*kT*, the leading order behaviour is

4.4 $$\begin{array}{rl} \alpha_{c} & \approx -\pi S_{33}k\int_{0}^{\sigma_{m}}\int_{-1/2d}^{1/2d}\sigma_{a}\left\{8\pi^{2}\delta \sigma_{a}^{4}\frac{\partial \delta }{\partial e_{zz}}+\frac{\sin^{2}(\pi d\sigma_{z})}{\rho \pi^{2}d^{2}}\frac{\partial C_{33}}{\partial e_{zz}}+\frac{\sigma_{a}^{2}}{\rho }\frac{\partial C_{44}}{\partial e_{zz}}\right\}\\ & \;\times \left(4\pi^{2}\delta^{2}\sigma_{a}^{4}+\frac{C_{33}}{\rho \pi^{2}d^{2}}\sin^{2}(\pi d\sigma_{z})+\frac{C_{44}\sigma_{a}^{2}}{\rho }\right)d\sigma_{z}\;d\sigma_{a}. \end{array}$$

We see that as the temperature tends to zero the thermal expansion coefficient exponentially approaches zero, whereas at high temperatures the thermal expansion coefficient loses all temperature dependence and becomes simply a constant.

The sensitivities of the predictions of equation (4.2) to the differentials with respect to strain of *δ*, *C*_33_ and *C*_44_ are given in appendix B. Doubling ∂*δ*/∂*e*_*zz*_ has a significant effect on the prediction but the effect of halving the value is much less, i.e. the influence is nonlinear. Changing ∂*C*_33_/∂*e*_*zz*_ by ±1 × 10^11^ Pa (a few per cent) increases/decreases the prediction by the same amount, whereas increasing the value of ∂*C*_44_/∂*e*_*zz*_ by a factor of 4 increases the prediction significantly; however, reducing it by a factor 10 makes only a small difference, i.e. again the influence is nonlinear.

## 5. Coefficient of thermal expansion parallel to the basal plane—*α*_*a*_

The CTE, *α*_*a*_, is based on equation (2.12). As before, in deriving an expression for the CTE perpendicular to the basal plane, the second term in equation (2.12) can be ignored by reasoning that *S*_13_ is relatively small, thereby making this term insignificant. Thus, the thermal strains within the basal planes are assumed to be independent of those between layers.

Thus equation (2.12) becomes

5.1 $$\alpha_{a}=(S_{11}+S_{12}){∭}_{BZ}\sum_{p}k{\left(\frac{hv_{p}}{kT}\right)}^{2}\frac{\exp(hv_{p}/kT){\left[\gamma_{p}\right]}_{xx}}{{\left\{\exp(hv_{p}/kT)-1\right\}}^{2}}d^{3}\sigma .$$

Noting that the first terms inside the square brackets for the three vibrational modes given in equations (3.6) are dominant, it was therefore assumed that the other terms could be ignored. This assumption greatly simplifies the mathematics. The vibrational mode terms given by equations (3.6) become

5.2 $$\begin{array}{rl} v_{1} & =\sqrt{\frac{C_{11}}{\rho }}\sigma_{a},\\ v_{2} & =\sqrt{\frac{\left(C_{11}-C_{12}\right)}{2\rho }}\sigma_{a}\\ \;\mathrm{and}\;v_{3} & =2\pi \delta \sigma_{a}^{2}. \end{array}\}$$

The anharmonic terms can then be easily calculated for the three modes, giving

5.3 $$\begin{array}{rl} \;{\left[\gamma_{1}\right]}_{xx} & =-\frac{1}{v_{1}}\frac{\partial v_{1}}{\partial e_{xx}}=-\frac{1}{2C_{11}}\frac{\partial C_{11}}{\partial e_{xx}},\\ {\left[\gamma_{2}\right]}_{xx} & =-\frac{1}{v_{2}}\frac{\partial v_{2}}{\partial e_{xx}}=-\frac{1}{2(C_{11}-C_{12})}\frac{\partial (C_{11}-C_{12})}{\partial e_{xx}}\\ \;\mathrm{and}\;{\left[\gamma_{3}\right]}_{xx} & =-\frac{1}{v_{3}}\frac{\partial v_{3}}{\partial e_{xx}}=-\frac{1}{\delta }\frac{\partial \delta }{\partial e_{xx}}. \end{array}\}$$

The integral on the right-hand side of equation (5.1) is accomplished using various substitutions, defining Debye characteristic temperatures for the longitudinal, transverse and out-of-plane directions, *θ*_L_, *θ*_T_ and *θ*_o_, as

5.4 $$\begin{array}{rl} \frac{\theta_{L}}{T} & =x_{L}=\frac{h}{kT}\sqrt{\frac{C_{11}}{\rho }}\sigma_{m}=\frac{2562}{T},\\ \frac{\theta_{T}}{T} & =x_{T}=\frac{h}{kT}\sqrt{\frac{\left(C_{11}-C_{12}\right)}{2\rho }}\sigma_{m}=\frac{1650}{T}\\ \;\mathrm{and}\;\frac{\theta_{o}}{T} & =x_{o}=\frac{h}{kT}2\pi \delta \sigma_{m}^{2}=\frac{1120}{T}. \end{array}\}$$

Treating each term individually we have that

5.5 $$\alpha_{a}=\alpha_{a_{1}}+\alpha_{a_{2}}+\alpha_{a_{3}},$$

with the in-plane longitudinal modes

5.6 $$\alpha_{a_{1}}=(S_{11}+S_{12})k{\left(\frac{h}{kT}\right)}^{2}2\pi \int_{-1/2d}^{1/2d}d\sigma_{z}\int_{0}^{\sigma_{m}}\left(-\frac{\sigma_{a}^{3}}{2\rho }\;\frac{\partial C_{11}}{\partial e_{xx}}\;\frac{\exp(h/kT\sqrt{\left(C_{11}/\rho \right)}\sigma_{a})}{{\left\{\exp(h/kT\sqrt{C_{11}/\rho }\sigma_{a})-1\right\}}^{2}}\right)d\sigma_{a},$$

giving

5.7 $$\alpha_{a_{1}}=-\frac{\pi \rho k(S_{11}+S_{12})}{C_{11}d}{\left[\frac{kT}{h}\right]}^{2}\left\{\left(\frac{1}{C_{11}}\right)\frac{\partial C_{11}}{\partial e_{xx}}\right\}J_{3}\left(\frac{\theta_{L}}{T}\right).$$

The expression for the in-plane transverse modes, *α*_*a*_2__, is identical to the expression for *α*_*a*_1__, with *C*_11_ replaced with $C_{66}=\frac{1}{2}(C_{11}-C_{12})$,

5.8 $$\alpha_{a_{2}}=-\frac{\pi \rho k(S_{11}+S_{12})}{C_{66}d}{\left[\frac{kT}{h}\right]}^{2}\left\{\left(\frac{1}{C_{66}}\right)\frac{\partial C_{66}}{\partial e_{xx}}\right\}J_{3}\left(\frac{\theta_{T}}{T}\right).$$

For the out-of-plane transverse modes, we have

5.9 $$\alpha_{a_{3}}=(S_{11}+S_{12})k{\left(\frac{h}{kT}\right)}^{2}2\pi \int_{-1/2d}^{1/2d}d\sigma_{z}\int_{0}^{\sigma_{m}}\left(-4\pi^{2}\delta \sigma_{a}^{5}\frac{\partial \delta }{\partial e_{xx}}\frac{\exp(2\pi h\delta \sigma_{a}^{2}/kT)}{{\left\{\exp(2\pi h\delta \sigma_{a}^{2}/kT)-1\right\}}^{2}}\right)d\sigma_{a},$$

giving

5.10 $$\alpha_{a_{3}}=-\frac{k(S_{11}+S_{12})}{2d\delta }\left[\frac{kT}{h}\right]\left\{\left(\frac{1}{\delta }\right)\frac{\partial \delta }{\partial e_{xx}}\right\}J_{2}\left(\frac{\theta_{o}}{T}\right),$$

where *J*_3_(*θ*_*L*_/*T*), *J*_3_(*θ*_*T*_/*T*) and *J*_2_(*θ*_*o*_/*T*) are Debye integrals of order 3 and 2 which can be solved numerically as

5.11 $$J_{n}(x_{i})=\int_{0}^{x_{i}}\frac{t^{n}e^{t}}{{\left(e^{t}-1\right)}^{2}}\;dt.$$

It is possible to make the simplifying assumption [6] that including contributions from both the in-plane acoustic and the higher frequency optical mode can be made by extending the upper integral limit as follows:

5.12 $$\begin{array}{rl} \theta_{M} & =\sqrt{2}\theta_{L}=3623,\\ \theta_{Q} & =\sqrt{2}\theta_{T}=2334\\ \;\mathrm{and}\;\theta_{N} & =2\theta_{o}=2238. \end{array}\}$$

**Figure 5.**
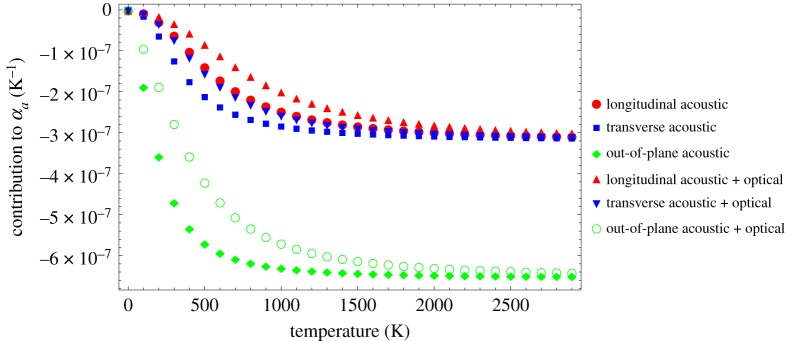
Contribution of each of the terms in equations (5.7), (5.8) and (5.10) to *α*_*a*_. (Online version in colour.)

The square root arises from the particular wavenumber–frequency relationships for the in-plane mode. The contributions that each of equations (5.7), (5.8) and (5.10) makes to the total CTE, assuming that *C*_11_^−1^∂*C*_11_/∂*e*_*xx*_ = *C*_66_^−1^∂*C*_66_/∂*e*_*xx*_ = *δ*^−1^∂*δ*/∂*e*_*xx*_ = 1, are given in figure 5. Solutions are included for acoustic (*θ*_*L*_, *θ*_*T*_, *θ*_*o*_) and acoustic plus optical (*θ*_*M*_, *θ*_*Q*_, *θ*_*N*_) modes.

**Figure 6.**
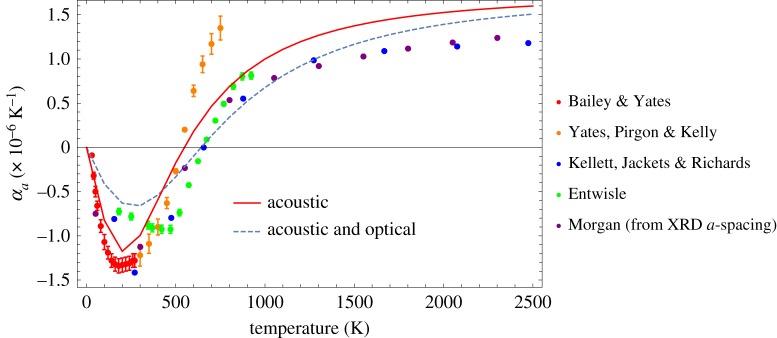
Predictions of the basal plane coefficient of thermal expansion *α*_*a*_ compared with experimental data; see references [27,31,34–36]. (Online version in colour.)

The resultant curves are slightly different from those previously published [6], which may be related to some errors in the previous equations. Generally, the contributions to *α*_*a*_ from the longitudinal and transverse modes are similar in magnitude, while the magnitude of the out-of-plane mode is larger, as would be expected. In all cases, the acoustic plus optical is smaller in magnitude in the lower to mid-temperature range (between 0 and ∼1500 K) than for the acoustic alone. This effect is larger in the case of the out-of-plane mode. These differences indicate that combining the optical and acoustic modes results in less excitation in the lower to mid-temperature ranges. Above ∼1500 K, the contributions from the two cases converge. This has implications for the prediction of the basal plane CTE *α*_*a*_ as discussed and shown in figure 6.

Kelly [6] derived values for terms *C*_11_^−1^∂*C*_11_/∂*e*_*xx*_, *C*_66_^−1^∂*C*_66_/∂*e*_*xx*_ and *δ*^−1^∂*δ*/∂*e*_*xx*_ based on assuming a Morse potential and ‘scientific judgement’ on coefficients as −8.2, −8.2 and 8.2, respectively; however, his calculations are not outlined in any great detail. In addition, there are typing errors and omissions in the version of equations (5.7), (5.8) and (5.10) given in [6]. In this paper, to obtain a reasonable fit by eye, it was found necessary to use values of −8.2, −8.2 and 5.47, respectively. Data from various references for *α*_*a*_, as discussed in §4 of this paper, are given in figure 6 along with the prediction of *α*_*a*_ obtained using equation (5.5).

Applying these values and summing the three expressions as equation (5.5), the following comparisons with experimental data are obtained.

The fit to the data in figure 6 is very similar to that given by Kelly [6], although there is significant scatter in the data; a much better experimental dataset would be required to justify further optimization of equation (5.5). The initial negative CTE at low temperatures arises from the interaction of the out-of-plane anharmonic modes with the two in-plane modes, whereas at temperatures above about 300 K the in-plane modes start to dominate and *α*_*a*_ becomes positive around 675 K. With reference to figure 5, making the approximation of ignoring the optical contribution gives a better fit to the data at low to mid-temperature ranges (between 0 and 1500 K) and makes little contribution to the higher temperatures, above 1500 K.

## 6. Graphite crystal CTE model—Lennard-Jones approach to deriving a relationship for *α*_*c*_

### (a) The relationship between lattice *d*-spacing and temperature

To develop the methodology further, a relationship between lattice *d*-spacing and temperature is required. This is obtained using fits to experimental data obtained from Nelson & Riley [29],

6.1 $$d=\frac{1}{2}(0.66915+18.7\times 10^{-6}(T+273)+1.263\times 10^{-9}{\left(T+273\right)}^{2}),$$

Matuyama [38]

6.2 $$d=0.33525+8.241\times 10^{-6}(T+273)+1.03\times 10^{-9}{\left(T+273\right)}^{2}$$

and Walker *et al*. [39]

6.3 $$d=0.3358+9.52\times 10^{-6}(T+273).$$

Taking the simple mean of the above fits gives

6.4 $$d=0.3328+8.63\times 10^{-6}T+5.538\times 10^{-10}T^{2}.$$

**Figure 7.**
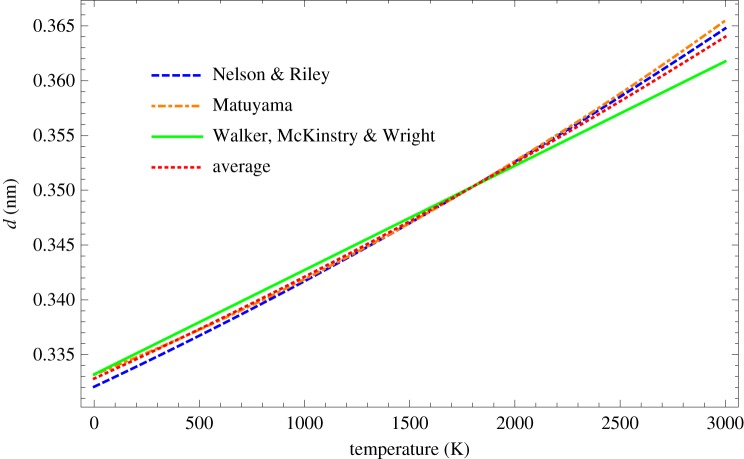
Fits to experimental data of lattice *d*-spacing as a function of temperature [29,33,38,39]. (Online version in colour.)

These fits are shown in figure 7. The average fit implies that the *d*-spacing at absolute zero and room temperature has values of 0.3328 nm and 0.3354 nm, respectively, compared with the usually accepted value of 0.335 nm at ambient room temperature [14,15]. It is of course possible to determine an equilibrium value of *d* from a calculation of the thermodynamically stable crystal structure. We, however, decided to take an average from experimental data because, as will become apparent later on, this lattice spacing temperature dependence is crucial for better matching to experimental data at high temperatures.

### (b) Lennard-Jones approach to deriving a relationship for *α*_*c*_

This approach is outlined in references [10–12] and starts with the Lennard-Jones-type relationship for the potential energy between two atoms separated by distance *d*_0_,

6.5 $$E^{\prime }=-A\left\{\frac{1}{r^{6}}-\frac{1}{2}\frac{r_{0}^{6}}{r^{12}}\right\},$$

where *r* is the atomic separation, *A* = 2.43 × 10^−78^ Jm^6^ and *r*_0_ is a constant.

An approximation is required in which the carbon atoms are assumed to be distributed with uniform density within a set of basal planes. This is achieved by integrating equation (6.5) over the graphite crystal lattice to give the equivalent energy per unit volume of the lattice. Following a methodology suggested by Crowell [40] gives

6.6 $$E=-2\pi \sigma AN_{0}\int_{0}^{\infty }\frac{x}{{\left(d^{2}+x^{2}\right)}^{3}}\;dx+\pi \sigma AN_{0}r_{0}^{6}\int_{0}^{\infty }\frac{x}{{\left(d^{2}+x^{2}\right)}^{6}}\;dx,$$

where *σ* = 1/*q* (*q* = 2.62 × 10^−20^ m^2^ is the area per atom in a basal layer [8]) and *N*_0_ = ^*ρN*_*a*_^/_*M*_ = 1.13 × 10^29^ atoms m^−3^ is the number of atoms per unit volume (graphite crystal density *ρ*_*c*_ = 2.26 g cm^−3^, Avogadro's number *N*_*a*_ = 6.022 × 10^23^ atoms or molecules per gram-mole, the molecular weight of carbon *M* = 12.01 atomic mass units). The radial distance in the basal planes is given by *x*.

Solving equation (6.6) gives

6.7 $$E=\frac{\pi \sigma AN_{0}}{2d^{4}}\left[\frac{1}{5}{\left(\frac{r_{0}}{d}\right)}^{6}-1\right].$$

The equilibrium spacing, *d*_0_, is given by ∂*E*/∂*d* = 0, which leads to

6.8 $$r_{0}^{6}=2d_{0}^{6}.$$

The elastic modulus for a lattice spacing, *d*, perpendicular to the basal plane is given by the second derivative of equation (6.7) with respect to strain between the basal planes, i.e.

6.9 $$C_{33}(d)=\frac{\partial^{2}E}{\partial e_{zz}^{2}}=d^{2}\left(\frac{\partial^{2}E}{\partial d^{2}}\right)=\frac{\pi \sigma AN_{0}}{2d^{4}}\left[22{\left(\frac{r_{0}}{d}\right)}^{6}-20\right],$$

which gives an equilibrium value of

6.10 $$C_{33}(d_{0})=\frac{12\pi \sigma AN_{0}}{d_{0}^{4}}.$$

Another important function is the derivative of the elastic modulus with respect to strain,

6.11 $$\frac{\partial C_{33}}{\partial e_{zz}}=d^{3}\frac{\partial^{3}E}{\partial d^{3}}=\frac{\pi \sigma AN_{0}}{d^{4}}\left[60-132{\left(\frac{r_{0}}{d}\right)}^{6}\right],$$

with an equilibrium value of

6.12 $${\left(\frac{\partial C_{33}}{\partial e_{zz}}\right)}_{d_{0}}=-\frac{204\pi \sigma AN_{0}}{d_{0}^{4}}=-17C_{33}(d_{0}).$$

**Figure 8.**
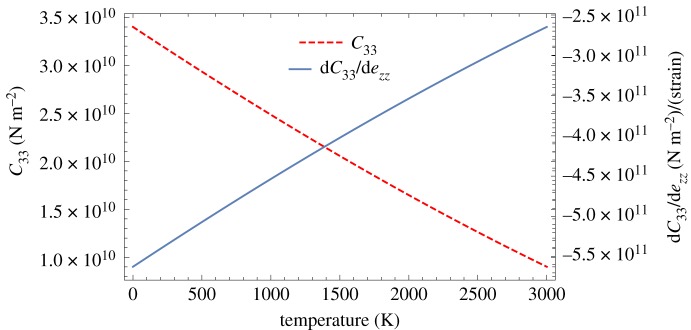
Modulus perpendicular to the basal plane, *C*_33_, and its derivative, ∂*C*_33_/∂*e*_*zz*_, as a function of temperature. (Online version in colour.)

Using the average relationship between temperature and lattice spacing *d*, given by equation (6.4), the relationships given by equations (6.9) and (6.11) are shown in figure 8. Thus we obtain room temperature values for *C*_33_ and ∂*C*_33_/∂*e*_*zz*_ of 31.25 GPa and −531.7 GPa, respectively, which are slightly lower than the accepted value of 36.5 GPa [15] for *C*_33_ and −600.0 GPa for ∂*C*_33_/∂*e*_*zz*_ used by Kelly & Walker [9].

If the crystal lattice is expanded from *d*_0_ to some other spacing *d*, equation (6.7) becomes

6.13 $$E=\frac{C_{33}(d_{0})}{24}\left[\frac{2}{5}{\left(\frac{d_{0}}{d}\right)}^{10}-{\left(\frac{d_{0}}{d}\right)}^{4}\right].$$

Thus, equation (2.4) can be modified to

6.14 $$F=U_{0}+\frac{C_{33}(d_{0})}{24}\left[\frac{2}{5}{\left(\frac{d_{0}}{d}\right)}^{10}-{\left(\frac{d_{0}}{d}\right)}^{4}\right]+kT{∭}_{BZ}\sum_{p}\ln\left[1-\exp\left(-\frac{hv_{p}}{kT}\right)\right]d^{3}\sigma .$$

Minimizing equation (6.14) gives

6.15 $$\frac{\partial F}{\partial d}=\frac{C_{33}(d_{0})}{6}\left[-\frac{d_{0}^{10}}{d^{11}}+\frac{d_{0}^{4}}{d^{5}}\right]-\frac{1}{d}{∭}_{BZ}\sum_{p}\frac{hv_{p}}{\exp(hv_{p}/kT)-1}{\left[\gamma_{p}\right]}_{zz}d^{3}\sigma =0.$$

Noting that *α*_*c*_ = (1/*d*)(∂*d*/∂*T*) and pre-multiplying by *d* before differentiating equation (6.15) with respect to *T* gives

6.16 $$\frac{C_{33}(d_{0})}{6}\left[10{\left(\frac{d_{0}}{d}\right)}^{10}-4{\left(\frac{d_{0}}{d}\right)}^{4}\right]\alpha_{c}={∭}_{BZ}\sum_{p}k{\left(\frac{hv_{p}}{kT}\right)}^{2}\frac{\exp\left(hv_{p}/kT\right){\left[\gamma_{p}\right]}_{zz}}{{\left\{\exp(hv_{p}/kT)-1\right\}}^{2}}d^{3}\sigma .$$

As previously discussed, in the case of deriving *α*_*c*_ only the out-of-plane mode *v*_3_ is required. It can also be argued [12] that the only significant term in the vibrational mode is the first one, whereas the only significant term in the anharmonic term is the second one. This was justified by stating that previous numerical modelling showed this to be true for the first model [11], meaning it is reasonable that this would still hold true in this new approach. It should be noted that this approximation cannot hold to zero wavenumber, as will be shown later. Applying this reasoning, equations (3.6) and (3.7) simplify to

6.17 $$\begin{array}{rl} v_{3} & =2\pi \delta \sigma_{a}^{2}\\ \;\mathrm{and}\;{\left[\gamma_{3}\right]}_{zz} & =-\frac{\sin^{2}(\pi d_{0}\sigma_{z})}{8\rho \pi^{4}\delta^{2}d_{0}^{2}\sigma_{a}^{4}}\frac{\partial C_{33}}{\partial e_{zz}}. \end{array}\}$$

This simplifies equation (6.16) upon rearranging to

6.18 $$\alpha_{c}=-\frac{6}{C_{33}(d_{0})}\frac{\partial C_{33}}{\partial e_{zz}}{\left[10{\left(\frac{d_{0}}{d}\right)}^{10}-4{\left(\frac{d_{0}}{d}\right)}^{4}\right]}^{-1}\frac{h}{8\rho \pi^{2}\delta d_{0}^{3}}\frac{1}{T}\int_{0}^{x_{m}}\frac{\exp(x)}{{\left(\exp(x)-1\right)}^{2}}dx,$$

with an upper integration limit defined as

6.19 $$x_{m}=\frac{\theta_{o}}{T}=\frac{2\pi h\delta \sigma_{m}^{2}}{kT}=\frac{1120}{T}.$$

The version of equation (6.18) derived [12] mistakenly has a denominator of 4 instead of 8; however, this mistake is compensated for by an error in the lower integration limit as discussed below. The integral in equation (6.18) does not converge for small values of *x*—the neglected frequency terms in the approximation (equation (6.17)) are important in the small wavenumber regime—it is therefore necessary to define a lower limit. It was originally assumed [11] that the smallest value of *σ*_*a*_ at which the out-of-plane vibrations can be regarded as purely two dimensional is at the point where the first term starts to become significant and equal to the second term, leading to

$$\frac{C_{33}(d_{0})}{\rho d_{0}^{2}\pi^{2}}=4\pi^{2}\delta^{2}\sigma_{o}^{4},$$

giving

$$\sigma_{o}^{2}=\frac{1}{2\pi^{2}d_{0}\delta }\sqrt{\frac{C_{33}(d_{0})}{\rho }}$$

and

6.20 $$x_{o}=\frac{\theta^{\prime }}{T}=\frac{2\pi h\delta \sigma_{o}^{2}}{kT}=\frac{169.61}{T}.$$

However, this lower limit underestimates the value of the Debye integral derived using the full expressions for the out-of-plane vibration and its anharmonic by a factor of approximately 2. A lower limit of *x*_*o*_ ≈ 0.083*x*_*m*_, *θ*′ ≈ 93 K is required to give a good fit to available data. A smaller limit will overestimate the Debye integral whereas a higher limit will underestimate the Debye integral.

Equation (6.11) can be written in terms of equilibrium values as

6.21 $$\frac{\partial C_{33}}{\partial e_{zz}}=-\frac{1}{34}{\left(\frac{\partial C_{33}}{\partial e_{zz}}\right)}_{d_{0}}\left[10{\left(\frac{d_{0}}{d}\right)}^{4}-44{\left(\frac{d_{0}}{d}\right)}^{10}\right].$$

Equation (6.18) can now be solved to give

**Figure 9.**
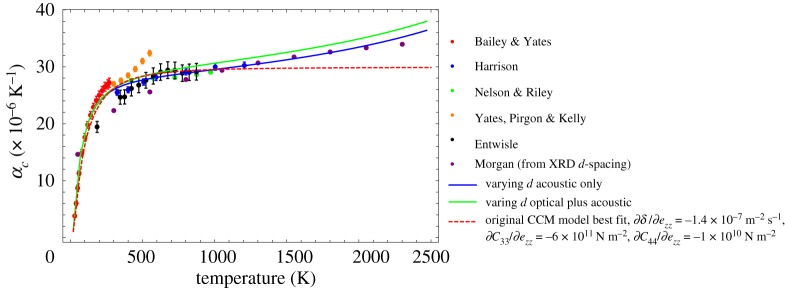
Prediction of *α*_*c*_ taking account of the change in *d*-spacing with increasing temperature, compared with experimental data [27,29,34–37] and previous model (equation (4.2)). (Online version in colour.)

6.22 $$\begin{array}{r} \alpha_{c}=\frac{1}{C_{33}(d_{0})}{\left(\frac{\partial C_{33}}{\partial e_{zz}}\right)}_{d_{0}}\left(\frac{6}{34}\right)\frac{\left[10-44{\left(d_{0}/d\right)}^{6}\right]}{\left[10{\left(d_{0}/d\right)}^{6}-4\right]}\frac{h}{8\rho \pi^{2}\delta d_{0}^{3}}\frac{1}{T}\left\{\frac{\exp(\theta_{o}/T)-\exp(\theta^{\prime }/T)}{\left[1-\exp(\theta_{o}/T)][1-\exp(\theta^{\prime }/T)\right]}\right\}, \end{array}$$

which is plotted in figure 9 along with the previous model given by equation (4.2) for reference. As before an attempt to include optical modes is included [6], by taking an upper limit of 2*θ*_*o*_.

We can again examine the behaviour of the thermal expansion coefficient in the high and low temperature limits by examining the leading order behaviour for *θ*′, *θ*_0_≫*T* and *θ*′, *θ*_0_≪*T*. It is important to remember that *θ*_*o*_ > *θ*′.

For low temperatures, *θ*′, *θ*_0_≫*T*, the leading order behaviour is

6.23 $$\alpha_{c}=\frac{1}{C_{33}(d_{0})}{\left(\frac{\partial C_{33}}{\partial e_{zz}}\right)}_{d_{0}}\left(\frac{6}{34}\right)\frac{\left[10-44{\left(d_{0}/d\right)}^{6}\right]}{\left[10{\left(d_{0}/d\right)}^{6}-4\right]}\frac{h}{8\rho \pi^{2}\delta d_{0}^{3}}\frac{1}{T}\left\{\exp\left(-\frac{\theta^{\prime }}{T}\right)-\exp\left(-\frac{\theta_{o}}{T}\right)\right\}.$$

For high temperatures, *θ*′, *θ*_0_≪*T*, the leading order behaviour is

6.24 $$\alpha_{c}=\frac{1}{C_{33}(d_{0})}{\left(\frac{\partial C_{33}}{\partial e_{zz}}\right)}_{d_{0}}\left(\frac{6}{34}\right)\frac{\left[10-44{\left(d_{0}/d\right)}^{6}\right]}{\left[10{\left(d_{0}/d\right)}^{6}-4\right]}\frac{h}{8\rho \pi^{2}\delta d_{0}^{3}}\left(\frac{\theta_{o}-\theta^{\prime }}{\theta_{o}\theta^{\prime }}\right).$$

We again see that, as the temperature tends to zero, the thermal expansion coefficient exponentially approaches zero. However, at high temperatures, the thermal expansion coefficient only has temperature dependence through the change in lattice spacing, *d*, with temperature. This temperature dependence is responsible for the up-turn of the CTE at high temperatures, which appears to be in good agreement with the data. Without it, the CTE would level off at high temperatures just like the original continuum coefficient of thermal expansion model.

Equations (5.5), (4.2) and (6.22) give methods of estimating crystal *α*_*a*_ and *α*_*c*_, respectively, from first principles without resorting to atomistic modelling. Furthermore, they allow for sensitivity studies to be performed to determine the influence of crystal strain, modulus, temperature, etc. on CTE. The sensitivity of this simplified methodology is mainly dependent on the ratio *C*^−1^_33_(*d*_0_)(∂*C*_33_/∂*e*_*zz*_)_*d*_0__, which is (from equation (6.12)) equal to −17. Kelly & Duff [8] calculated this ratio using a slightly different methodology based on the work of Agranovich & Semenov [41] and Girifalco & Lad [42] to be −7.27 × 10^11^/38.6 × 10^9^ =  − 18.8 and −6.04 × 10^11^/36.5 × 10^9^ =  − 16.5, respectively. Thus, the first ratio of 18.8 would overpredict *α*_*c*_ by 10% and the second ratio would underpredict by 3%.

## 7. Influence of loading polycrystalline graphite on individual crystal thermal expansion

It has been observed that both mechanical applied loading of unirradiated polycrystalline graphite [2] and irradiation creep strain [43] can induce a significant change in bulk CTE, with tensile strains reducing CTE and compressive strains increasing CTE. The equation for *α*_*c*_ derived above can be used to investigate the effect of pressure on the thermal expansion coefficients of graphite crystals [7], assuming a change in interlayer spacing. More recently, Marrow *et al.* [44] measured the change in lattice *d*-spacing with loading using neutron diffraction and synchrotron XRD. Taking the room temperature lattice *d*-spacing from [44] to be 0.3353 nm implies a maximum strain of ∼0.015% associated with a bulk stain of 3000 μ*ϵ*. Assuming a Young's modulus of 11.9 GPa [44], this implies a bulk stress loading of 35 MPa, which is a similar order to that given in [2].

Modifying equation (6.22) to be a function of strain gives

7.1 $$\begin{array}{rl} \alpha_{c} & =\frac{1}{C_{33}(d_{0})}{\left(\frac{\partial C_{33}}{\partial e_{zz}}\right)}_{d_{0}}\left(\frac{6}{34}\right)\frac{\left[10-44{\left(1/\epsilon_{PT}\right)}^{6}\right]}{\left[10\left(1/\epsilon_{PT}\right)6-4\right]}\frac{h}{8\rho \pi^{2}\delta d_{0}^{3}}\frac{1}{T}\\ & \;\times \left\{\frac{\exp(\theta_{o}/T)-\exp(\theta^{\prime }/T)}{\left[1-\exp(\theta_{o}/T)][1-\exp(\theta^{\prime }/T)\right]}\right\}, \end{array}$$

where

7.2 $$\epsilon_{PT}=\epsilon (T)+\epsilon (P),$$

**Figure 10.**
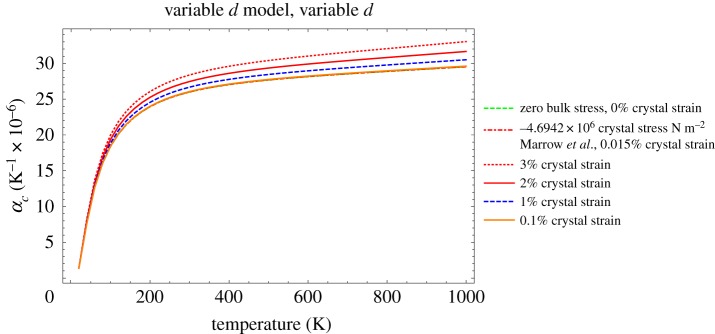
Prediction of *α*_*c*_, taking account of the change in *d*-spacing, with increasing temperature and differing amounts of crystal strain, note that the 0%, 0.015% (measured by Marrow *et al*. [44]) and 0.1% curves overlie each other. (Online version in colour.)

*ϵ*(*T*) is the lattice thermal strain, which can be calculated using equation (6.4), and *ϵ*(*P*) is the lattice strain due to bulk loading. Predictions of *α*_*c*_ are plotted in figure 10 over the range 20 to 1000 K.

From these calculations, it is clear that the change in crystal strain measured by Marrow *et al*. [44] is unlikely to account for the change in bulk CTE due to loading. It is also clear that crystal strains greater than approximately 1% are required to lead to significant changes in *α*_*c*_. Thus for practical purposes such strains are unlikely to be generated by bulk loading. However, they could result from fast neutron irradiation as discussed below.

## 8. Influence of irradiation on *α*_*a*_ and *α*_*c*_

**Figure 11.**
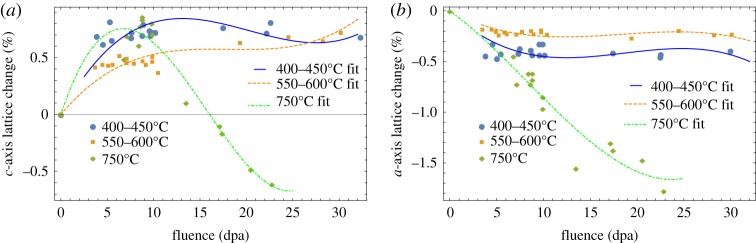
Irradiation-induced changes to the lattice *c*-spacing (*a*) and *a*-spacing (*b*), reported by the various authors from the UKAEA [45–47] and from NRG in Petten [48]. (Online version in colour.)

**Figure 12.**
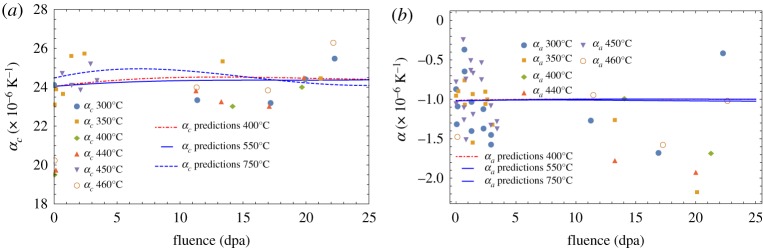
Thermal expansion coefficients both perpendicular (*a*) and parallel (*b*) to the basal plane measured on irradiated HOPG [1] and predictions using the NRG XRD data and equations (7.1) (*a*) and (5.5) (*b*). (Online version in colour.)

Small changes, less than a maximum of 2% at an irradiation fluence of 25 dpa, in lattice spacing due to fast neutron irradiation measured using XRD have been reported by the United Kingdom Atomic Energy Authority (UKAEA) [45–47] and more recently by the Nuclear Research Group (NRG) in Petten, The Netherlands [48]; see figure 11. These data have been used here in equations (5.5) and (7.1) to predict the change in CTE in the *a*- and *c*-directions as a function of fast neutron fluence and are compared with measurement of the thermal expansion measured on HOPG irradiated between 300°C and 650°C in figure 12. Although there is significant scatter in the measured data the predictions uphold the assumption of invariance in graphite crystal CTE for all practical purposes. However, data on irradiated polycrystalline graphite show a significant reduction in CTE with increasing fast neutron fluence. Possible reasons for this behaviour are discussed in §9.

## 9. Irradiation-induced changes to bulk polycrystalline CTE—application and some observations

To determine the bulk CTE, one can consider a simple Reuss model [49], in which all the crystals are arranged in series. For isotropic graphite, this gives a linear CTE of

9.1 $$\alpha_{\mathrm{bulk}}=\frac{1}{3}(\alpha_{c}+2\alpha_{a}).$$

**Figure 13.**
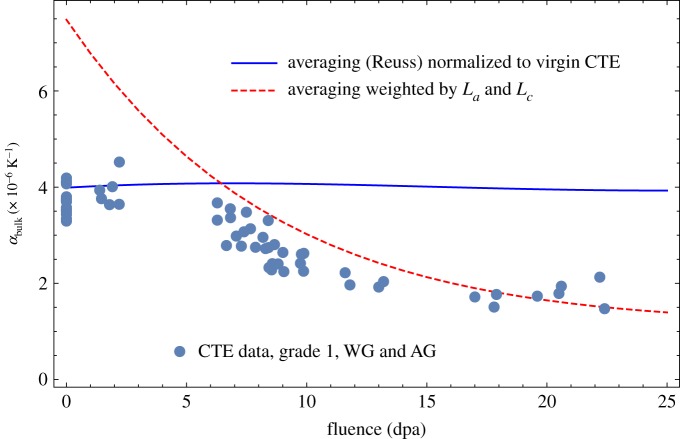
Predictions of bulk CTE compared with experimental data [48] for grade 1 graphite. (Online version in colour.)

If all the crystallites in nuclear graphite were randomly distributed with no porosity, this would imply an unirradiated CTE of ∼8 × 10^−6^ K^−1^. However, there is porosity particularly perpendicular to the crystal *c*-axis, which reduces the influence of the crystal *c*-axis expansion, reducing the overall bulk CTE. The expansion in the *c*-direction is said to fill this accommodation porosity without increasing the specimen length in that direction. Thus, using the predictions of crystal CTE in equation (9.2) and normalizing the results to the virgin value a prediction of irradiated bulk CTE is compared with the measured data for well-graphitized, medium-grained nuclear graphite grade 1 in figure 13.

**Figure 14.**
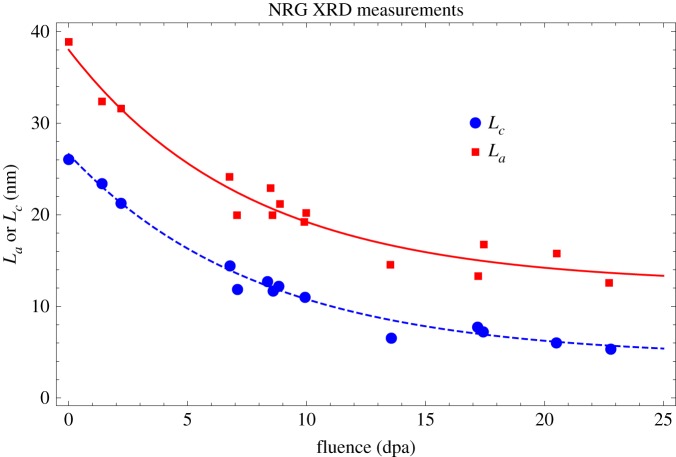
Grade 1 graphite irradiated at 750°C, change in crystal size *L*_*a*_ and *L*_*c*_ [48,51]. (Online version in colour.)

While this does predict some reduction in CTE with increasing fluence as observed in all medium-grained graphite, the reduction is small compared with the experimental observations. The Reuss approximation effectively assumes that the phases are infinite in one direction or are at least continuously connected in one direction. However, analysis of the XRD peak broadening, based on the Scherrer equation [50], showed that the crystal size *L*_*a*_ and *L*_*c*_ both decreased with increasing irradiation, appearing to start to saturate at higher fluence [48,51]; this is shown in figure 14. This may suggest that the Reuss approximation breaks down in much the same way as it does in composite mechanics with discontinuous reinforcements [52–54].

Assuming that the reduction in crystallite size increases porosity these data can be used to try and account for the increase in porosity by weighting equation (9.1) as follows:

9.2 $$\alpha_{\mathrm{bulk}}=\frac{1}{3}\left(\alpha_{c}\frac{L_{c}}{L_{c}(0)}+2\alpha_{a}\frac{L_{a}}{L_{a}(0)}\right),$$

where *L*_*c*_(0) and *L*_*a*_(0) are the unirradiated values of *L*_*c*_ and *L*_*a*_.

**Figure 15.**
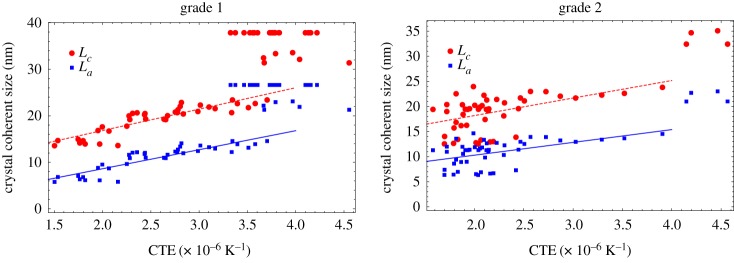
Measurements of crystal coherent scattering domain size against irradiated CTE for grade 1 and grade 2 graphite [48,51]. (Online version in colour.)

Figure 13 suggests a combination of the two models is required to fit the data, above and below about 5 dpa. Below 5 dpa bulk CTE is largely invariant to fast neutron fluence, whereas above 5 dpa CTE falls with increasing fluence. Figure 15 shows the crystal coherent scattering domain size data [48,51] against irradiated CTE for EU medium-grained nuclear graphite grade 1 and grade 2, clearly showing a correlation between CTE and large coherence crystal size. These comparisons suggest that the reason for the fall in CTE above 5 dpa is caused by irradiation-induced changes in crystal perfection, leading to disruption of the crystal lattice, and resulting in a smaller scattering length.

## 10. Conclusion

— Theoretical models for the prediction of the CTE in graphite crystals and HOPG proposed in the 1970s and 1980s have been revisited. Numerous errors have been corrected and equations reformulated where necessary.
— Using more recent input data, the equations have then been applied to the prediction of graphite crystal CTE both parallel and perpendicular to the hexagonal basal planes as a function of temperature, from absolute zero to 3000 K. The results are shown to give an excellent fit to experimental data.
— The theoretical equations have been reformulated to give the change in CTE as a function of strain. Recent neutron and XRD experiments measuring changes to lattice spacing under load have demonstrated that, in bulk graphite, crystal CTE is unlikely to be modified under load. This implies that changes in bulk graphite CTE observed in loaded graphite are most likely to be due to changes in microstructural perfection and orientation.
— The equations have then been applied to the prediction of crystal CTE subject to fast neutron irradiation showing that above 300°C the crystal CTE is largely invariant of fluence. This confirms experimental observations obtained on irradiated HOPG.
— The use of averaging techniques (Reuss) to predict irradiated induced bulk CTE in polycrystalline graphite is shown to give poor results. However, the fall in CTE at moderate to high irradiation fluence is shown to be proportional to the crystal coherent scattering domain size. This may imply that changes to irradiated CTE may be driven by the formation of nano-cracks or that there exists a length below which the Reuss approximation is invalid. This would also imply that irradiation-induced dimensional changes and modulus changes are driven by larger microstructural features.

## Figures and Tables

**Figure 16.**
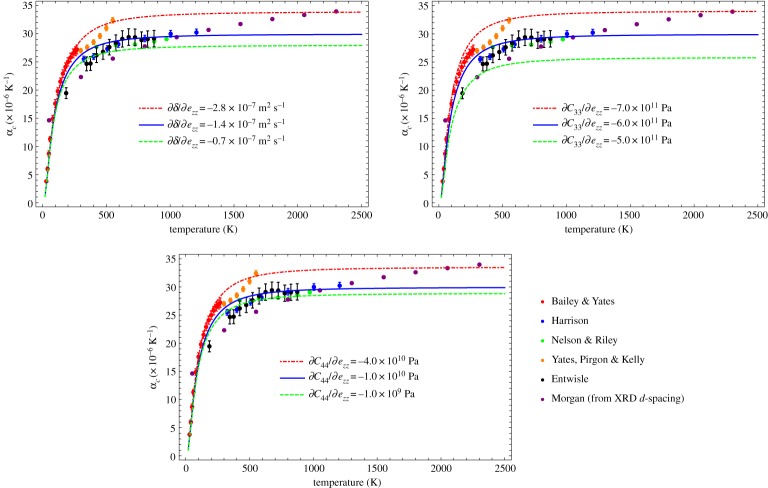
Sensitivity of *α*_*c*_ to ∂*C*_33_/∂*e*_*zz*_, ∂*C*_44_/∂*e*_*zz*_ and ∂*δ*/∂*e*_*zz*_ [27,29,34–37].

**Figure 17.**
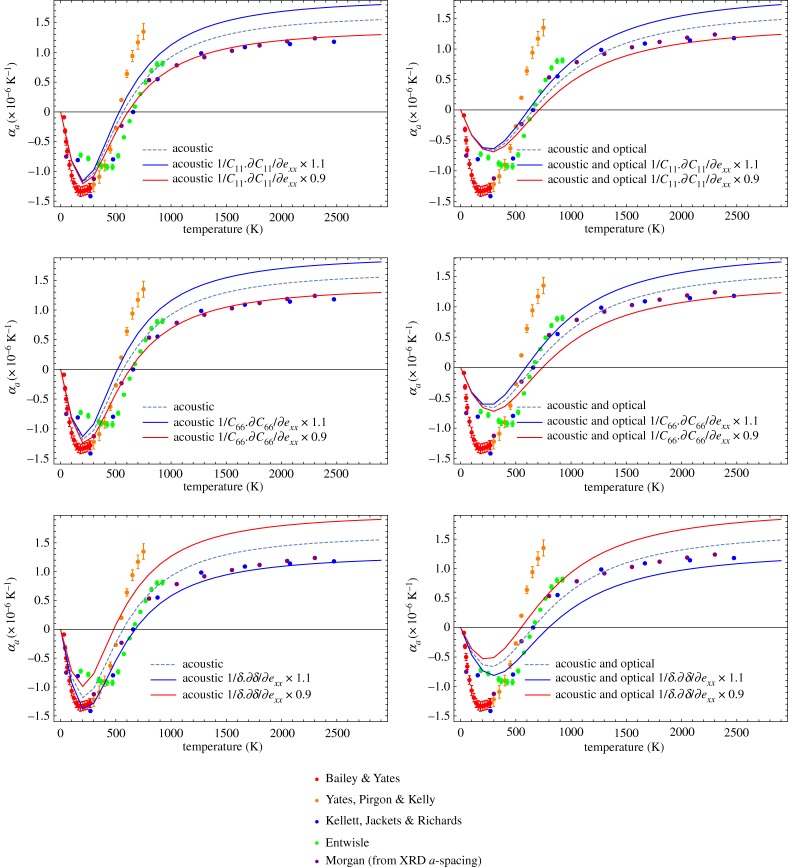
Sensitivity of *α*_*a*_ to simplified anharmonics *C*_11_^−1^∂*C*_11_/∂*e*_*xx*_, *C*_66_^−1^∂*C*_66_/∂*e*_*xx*_ and *δ*^−1^∂*δ*/∂*e*_*xx*_ [27,31,34–36].

## Data accessibility

This article does not contain any additional data.

## Appendix A. Tables of data

See tables 1 and 2.

## Appendix B. Sensitivity studies

See figures 16 and 17.

## References

[1] Marsden BJ, Hall GN 2016 4.11 graphite in gas-cooled reactors BT—reference module in materials science and materials engineering. Amsterdam, The Netherlands: Elsevier (10.1016/B978-0-12-803581-8.00729-3)

[2] Preston SD, Marsden BJ 2006 Changes in the coefficient of thermal expansion in stressed Gilsocarbon graphite. Carbon N. Y. 44, 1250–1257. (10.1016/j.carbon.2005.10.045)

[3] Marsden BJ, Haverty M, Bodel W, Hall GN, Jones AN, Mummery PM, Treifi M 2016 Dimensional change, irradiation creep and thermal/mechanical property changes in nuclear graphite. Int. Mater. Rev. 61, 155–182. (10.1080/09506608.2015.1136460)

[4] Sutton AL, Howard VC 1962 The role of porosity in the accommodation of thermal expansion in graphite. J. Nucl. Mater. 7, 58–71. (10.1016/0022-3115(62)90194-0)

[5] Kelly BT 1982 Graphite—the most fascinating nuclear material. Carbon N. Y. 20, 2–11. (10.1016/0008-6223(82)90421-3)

[6] Kelly BT 1972 The thermal expansion coefficient of graphite parallel to the basal planes. Carbon N. Y. 10, 429–433. (10.1016/0008-6223(72)90059-0)

[7] Kelly BT 1981 The effect of pressure and fast neutron irradiation on the thermal expansion coefficients of graphite crystals. Carbon N. Y. 19, 71–73. (10.1016/0008-6223(81)90109-3)

[8] Kelly BT, Duff MJ 1970 On the validity of Lennard-Jones potentials for the calculation of elastic properties of a graphite crystal. Carbon N. Y. 8, 77–83. (10.1016/0008-6223(70)90130-2)

[9] Kelly BT, Walker PL 1970 Theory of thermal expansion of a graphite crystal in the semi-continuum model. Carbon N. Y. 8, 211–226. (10.1016/0008-6223(70)90116-8)

[10] Kelly BT 1971 A simple model of the effect of interstitial atoms on the interlayer properties of a graphite crystal. Carbon N. Y. 9, 627–631. (10.1016/0008-6223(71)90084-4)

[11] Kelly BT 1970 An approximate theory of the hexagonal axis expansion coefficient of imperfect graphite crystals. J. Nucl. Mater. 35, 51–54. (10.1016/0022-3115(70)90026-7)

[12] Kelly BT 1972 The high temperature thermal expansion of graphite parallel to the hexagonal axis. Carbon N. Y. 10, 435–438. (10.1016/0008-6223(72)90060-7)

[13] Kelly BT 1967 The effect of defects on the basal plane thermal conductivity of a graphite crystal. Carbon 5, 247–260. (10.1016/0008-6223(67)90006-1)

[14] Michel KH, Verberck B 2008 Theory of the elastic constants of graphite and graphene. Phys. Status Solidi B 245, 2177–2180. (10.1002/pssb.200879604)

[15] Savini G, Heggie M 2007 Mesoscale elastic constants in graphite. In *Carbon Conf. Seattle, WA, 15–20 July 2007*, paper D102. American Carbon Society, See http://acs.omnibooksonline.com.

[16] Zwikker C 1954 Physical properties of solid materials. London, UK: Pergamon Press.

[17] Chung DDL 2002 Review graphite. J. Mater. Sci. 37, 1–15. (10.5402/2012/852405)

[18] Grüneisen E 1912 Theorie des festen Zustandes einatomiger Elemente. Ann. Phys. 344, 257–306. (10.1002/andp.19123441202)

[19] Komatsu K, Nagamiya T 1951 Theory of the specific heat of graphite. J. Phys. Soc. Jpn. 6, 438–444. (10.1143/JPSJ.6.438)

[20] Komatsu K 1955 Theory of the specific heat of graphite II. J. Phys. Soc. Jpn. 10, 346–356. (10.1143/JPSJ.10.346)

[21] Love AE 1934 A treatise of the mathematical theory of elasticity, 4th edn Cambridge, UK: Cambridge University Press.

[22] Lord Rayleigh. 1937 The theory of sound, 2nd edn, vol. I, p. 352 London, UK: Macmillan Press.

[23] Bacon GE 1951 The interlayer spacing of graphite. Acta. Cryst. 4, 558–561. (10.1107/S0365110X51001781)

[24] Franklin RE 1951 The structure of graphitic carbons. Acta. Cryst. 4, 253–261. (10.1107/S0365110X51000842)

[25] Steward EG, Cook BP 1960 X-ray measurement of thermal expansion perpendicular to the layer planes of artificial and natural graphites. Nature 185, 78–80. (10.1038/185078b0)

[26] Steward EG, Cook BP, Kellett EA 1960 Dependence on temperature of the interlayer spacing in carbons of different graphitic perfection. Nature 187, 1015–1016. (10.1038/1871015a0)

[27] Morgan WC 1971 The thermal expansion coefficients of graphite crystals. Carbon 10, 72–79. (10.1016/0008-6223(72)90011-5)

[28] Kellett EA, Richards BP 1964 The thermal expansion of graphite within the layer planes. J. Nucl. Mat. 12, 184–192. (10.1016/0022-3115(64)90139-4)

[29] Nelson JB, Riley DP 1945 The thermal expansion of graphite from 15°C to 800°C: part I. Exp. Proc. Phys. Soc. 57, 477–486. (10.1088/0959-5309/57/6/303)

[30] Yang KT 1962 The determination of the interlayer spacings in carbons at high temperatures. In *Proc. 5th Carbon Conf.*, *University Park, PA, 19–23 June 1961*, vol. 1, pp. 492–496. New York, NY: Pergamon Press.

[31] Kellett EA, Jackets BP, Richards BP 1964 A study of the amplitude of vibration of carbon atoms in the graphite structure. Carbon 2, 175–183. (10.1016/0008-6223(64)90058-2)

[32] Baskin Y, Meyer L 1955 Lattice constants of graphite at low temperatures. Phys. Rev. 100, 544 (10.1103/PhysRev.100.544)

[33] Kelly BT 1981 Physics of graphite. London, UK: Applied Science Publishers.

[34] Entwisle F 1962 Thermal expansion of pyrolytic graphite. Phys. Lett. 2, 236–238. (10.1016/0031-9163(62)90243-3)

[35] Bailey AC, Yates B 1970 Anisotropic thermal expansion of pyrolytic graphite at low temperature. J. Appl. Phys. 41, 5088–5091. (10.1063/1.1658609)

[36] Yates B, Pirgon O, Kelly BT 1974 The anisotropic thermal expansion of graphite at elevated temperatures, paper 46. In *Proc. 4th SCI Conf. Industrial Carbons and Graphite, Imperial College London, UK, 23–27 September 1974*. London, UK: Society of Chemical Industry (SCI).

[37] Harrison JW 1977 Absolute measurements of the coefficient of thermal expansion of pyrolytic graphite from room temperature to 1200 K and a comparison with current theory. High Temp. High Press. 9, 211–219.

[38] Matuyama E 1954 A high-temperature, X-ray diffraction, powder camera. J Sci. Instrum. 32, 229–231. (10.1088/0950-7671/32/6/308)

[39] Walker PL, McKinstry HA, Wright CC 1953 X-ray diffraction studies of a graphitized carbon. Ind. Eng. Chem. 45, 1711–1715. (10.1021/ie50524a033)

[40] Crowell AD 1954 Approximate method of evaluating lattice sums of r-n for graphite. J. Chem. Phys. 22, 1397–1399. (10.1063/1.1740404)

[41] Agranovich VM, Semenov LP 1962 Contribution to the theory of radiation effects on some properties of graphite. Sov. J. At. Energy 10, 569–573. (10.1007/BF01591263)

[42] Girifalco LA, Lad RA 1956 Energy of cohesion, compressibility, and the potential energy functions of the graphite system. J. Chem. Phys. 25, 693–697. (10.1063/1.1743030)

[43] Gray BS, Brocklehurst JE, McFarlane AA 1967 The irradiation induced plasticity in graphite under constant stress. Carbon N. Y. 5, 173–180. (10.1016/0008-6223(67)90071-1)

[44] Marrow TJ *et al.* 2016 In situ measurement of the strains within a mechanically loaded polygranular graphite. Carbon N. Y. 96, 285–302. (10.1016/j.carbon.2015.09.058)

[45] Henson RW, Reynolds WN 1965 Lattice parameter changes in irradiated graphite. Carbon N. Y. 3, 277–287. (10.1016/0008-6223(65)90062-X)

[46] Goggin PR, Henson RW, Perks AJ, Reynolds WN 1964 Dimensional changes in the irradiated graphite lattice. Carbon N. Y. 1, 189–200. (10.1016/0008-6223(64)90075-2)

[47] Henson RW, Perks AJ, Simmons JHW 1968 Lattice parameter and dimensional changes in graphite irradiated between 300 and 1350 degrees C. Carbon N. Y. 6, 789–806. (10.1016/0022-3115(70)90026-7.)

[48] Vreeling JA, Smit-Groen VM 2011 X-ray diffraction experiments on irradiated graphite. In *Proc. INGSM-12, Jeju, South Korea*, 2011. See https://nucleus.iaea.org/Pages/nuclear-graphite-knowledge-base.aspx.

[49] Reynolds WN 1965 The mechanical properties of reactor graphite. Philos. Mag. 11, 357–368. (10.1080/14786436508221862)

[50] Patterson AL 1939 The Scherrer formula for X-ray particle size determination. Phys. Rev. 56, 978–982. (10.1103/PhysRev.56.978)

[51] Heijna MCR 2014 Report on XRD on low dose irradiation experiment at 750°C, INNOGRAPH-1C PIE report. EU Archer Report by NRG Petten D41.23. See http://www.archer-project.eu.

[52] Eshelby JD 1957 The determination of the elastic field of an ellipsoidal inclusion, and related problems. Proc. R. Soc. Lond. A 241, 376–396. (10.1098/rspa.1957.0133)

[53] Cox HL 1952 The elasticity and strength of paper and other fibrous materials. Br. J. Appl. Phys. 3, 72–79. (10.1088/0508-3443/3/3/302)

[54] Halpin JC, Kardos JL 1972 Moduli of crystalline polymers employing composite theory. J. Appl. Phys. 43, 2235–2241. (10.1063/1.1661482)

[55] Kelly B 1975 The basal thermal expansion of graphite and a relationship between the bond bending resistance and the in-plane elastic constants. Carbon N. Y. 13, 350 (10.1016/0008-6223(75)90043-3)

[56] Kelly B 1973 Effects of the bond-bending coefficient on the thermal expansion coefficient of graphite parallel to the hexagonal axis. Carbon N. Y. 11, 379–381. (10.1016/0008-6223(73)90077-8)

